# Evolutionary convergence on hummingbird pollination in Neotropical *Costus* provides insight into the causes of pollinator shifts

**DOI:** 10.1111/nph.18464

**Published:** 2022-09-24

**Authors:** Kathleen M. Kay, Dena L. Grossenbacher

**Affiliations:** ^1^ Department of Ecology and Evolutionary Biology University of California, Santa Cruz Santa Cruz CA 95060 USA; ^2^ Department of Biology California Polytechnic State University San Luis Obispo CA 93401 USA

**Keywords:** convergent evolution, *Costus*, floral traits, hummingbird pollination, orchid bees, pollination syndromes, tropical biology

## Abstract

- The evolution of hummingbird pollination is common across angiosperms throughout the Americas, presenting an opportunity to examine convergence in both traits and environments to better understand how complex phenotypes arise. Here we examine independent shifts from bee to hummingbird pollination in the Neotropical spiral gingers (*Costus*) and address common explanations for the prevalence of transitions from bee to hummingbird pollination.
- We use floral traits of species with observed pollinators to predict pollinators of unobserved species and reconstruct ancestral pollination states on a well‐resolved phylogeny. We examine whether independent transitions evolve towards the same phenotypic optimum and whether shifts to hummingbird pollination correlate with elevation or climate.
- Traits predicting hummingbird pollination include small flower size, brightly colored floral bracts and the absence of nectar guides. We find many shifts to hummingbird pollination and no reversals, a single shared phenotypic optimum across hummingbird flowers, and no association between pollination and elevation or climate.
- Evolutionary shifts to hummingbird pollination in *Costus* are highly convergent and directional, involve a surprising set of traits when compared with other plants with analogous transitions and refute the generality of several common explanations for the prevalence of transitions from bee to hummingbird pollination.

## Introduction

Convergent evolution of functionally similar phenotypes allows insight into the causes, constraints, repeatability and reversibility of adaptive evolution (Losos, 2011). When complex functions or ecological interactions evolve repeatedly, we can investigate whether the derived phenotypes are similar and whether certain aspects of the environment consistently drive their evolution (Donoghue, 2005; Collar *et al*., 2014). Moreover, when functional convergence is examined both within and among clades, we can tease apart the traits and environments commonly required vs those that are lineage‐specific (Gould, 2002).

The evolution of pollination syndromes in flowering plants provides just such a situation, with many independent transitions to phenotypically similar flowers that correspond to major functional groups of pollinators (Fenster *et al*., 2004). Hummingbird pollination has evolved many times in the Americas (Grant & Grant, 1968; Rosas‐Guerrero *et al*., 2014), with thousands of plant species from 68 families and 404 genera relying on hummingbirds for pollination (Abrahamczyk & Kessler, 2015). Hummingbird flowers often display a combination of traits thought to promote hummingbirds and deter bees (Grant & Grant, 1968; Schemske & Bradshaw, 1999; Castellanos *et al*., 2004; Bergamo *et al*., 2016). Copious and dilute nectar and a narrow shape, fitting a bird bill, are thought to promote effective hummingbird pollination, whereas red coloration, an elongated floral tube, and the lack of nectar guides, a landing platform and scent are thought to deter bees.

Comparative phylogenetic studies of the evolution of hummingbird pollination have shown convergence on a hummingbird syndrome both among distantly related plants and within young clades. One common finding is that hummingbird pollination is often a derived condition, most commonly from a bee‐pollinated ancestor, and reversals are rare (reviewed in Grant & Grant, 1968; Rosas‐Guerrero *et al*., 2014; Abrahamczyk & Renner, 2015). There are many hypotheses for this directional bias, but a well‐supported consensus remains elusive. Hummingbird pollination may generally be more effective than bee pollination, perhaps because hummingbirds do not consume pollen and tend to disperse pollen long distances (Thomson & Wilson, 2008; Krauss *et al*., 2017). There may be a bias towards the evolution of longer flowers because long flowers promote more efficient pollen transfer by pollinators accessing a floral tube for nectar (Darwin, 1862), and hummingbird bills are typically longer than bee proboscides (Whittall & Hodges, 2007). It might be that hummingbirds explore and visit nectar‐producing flowers regardless of whether they present a ‘hummingbird‐adapted’ phenotype, and then plants sometimes adapt to be pollinated by these common secondary pollinators (Grant & Grant, 1968; Thomson & Wilson, 2008; Rosas‐Guerrero *et al*., 2014). By contrast, bees may be excluded from visiting flowers by inconspicuous color signals and the lack of a landing area (Schemske & Bradshaw, 1999). Transitions to hummingbird pollination, which often involve acquiring red flower color, also may be promoted by the higher likelihood of structural mutations in the anthocyanin biosynthetic pathway causing violet to red color transitions compared to the reverse (Rausher, 2008). Finally, the more recent evolutionary origin and diversification of hummingbirds compared to pollinating bees may mean that there has simply been less time for reversals to accumulate (Grant & Grant, 1968). None of these explanations are mutually exclusive, and all would benefit from more detailed comparative work from both ecological and genetic perspectives across plant lineages.

In addition to insights from phylogenetic comparative approaches, understanding convergence on hummingbird pollination has benefitted from a move away from typological treatment of pollination syndromes, the suites of floral traits associated with particular groups of pollinators (Waser *et al*., 2011). Whereas some early work treated syndromes categorically, recent work has taken more quantitative approaches (Muchhala, 2006; Lagomarsino *et al*., 2017; Smith & Kriebel, 2018; reviewed in Dellinger, 2020; Bilbao *et al*., 2021). Rich data on floral phenotypes, including morphology, scent, reward and color, can reveal important variation among clades and geographic regions in the traits involved in pollination syndromes. This variation in turn can point to differences in selection pressures, such as in the abiotic environment or the behavior of specific hummingbird pollinators, as well as clade‐specific constraints on floral evolution (e.g. Muchhala *et al*., 2014; Bilbao *et al*., 2021).

Moreover, much recent work has evaluated the ability of pollination syndromes to predict actual pollinators in the field, as traits associated with syndromes may reflect a history of evolutionary selection but not a current reality of more generalized ecological interactions (Fenster *et al*., 2004). In a recent meta‐analysis, Rosas‐Guerrero *et al*. (2014) found that bird and bee pollination syndromes predicted the most effective pollinators well, but that predictability of syndromes was higher in tropical vs extratropical plant lineages. More studies that match floral traits with field observations of pollinators in a variety of clades and geographic locations will allow us to answer some of the longstanding questions raised by Grant & Grant (1968), such as: Which traits are consistently involved in evolutionary shifts from bee to hummingbird pollination, and which traits are lineage‐specific? Are intermediate floral phenotypes indicative of more recent evolutionary changes? Are interactions with secondary pollinators associated with syndrome transitions?

With detailed studies of both plant–pollinator interactions and traits, we can also assess environmental conditions involved in transitions to hummingbird pollination. Although the evolution of hummingbird pollination is a pervasive occurrence across the Americas (Grant & Grant, 1968; Stiles, 1978a; Abrahamczyk & Renner, 2015), there are different hypotheses about ecological drivers across biomes. In the tropics, cool, wet montane environments in which bee activity decreases are thought to select for transitions to hummingbird pollination (Cruden, 1972; Gentry, 1982; Kay *et al*., 2005; Dellinger *et al*., 2021). By contrast, Grant & Grant (1968) described hummingbird pollination in temperate North America as associated with moderate climates and habitats facilitating winter and spring hummingbird breeding (generally at lower elevations). Recent work on *Penstemon* supports this idea, with hummingbird pollination evolving at lower elevations and latitudes (Hamilton & Wessinger, 2022). Moreover, the diversity of resident tropical hummingbirds and competition among plants for pollinator services may lead to a broad range of hummingbird flower phenotypes (Grant & Grant, 1968; Gentry, 1982; Muchhala *et al*., 2014), whereas Grant & Grant (1968) hypothesized that in temperate areas, tight convergence on red flower color and a similar shape and size would be favored for plants using the few species of primarily migratory hummingbirds. Species‐level phylogenetic studies of transitions to hummingbird pollination, along with ecological niche and field pollination data, are necessary to examine these hypothesized differences between tropical and temperate regions (Dellinger *et al*., 2021, 2022a; Hamilton & Wessinger, 2022).

Here we examine pollinator visitation and floral evolution in the spiral ginger genus *Costus* (Costaceae), which has undergone multiple independent evolutionary transitions from bee to hummingbird pollination since establishing in the Neotropics *c*. 3 million years ago (Ma), as shown by a recent species‐level phylogenomic study with nearly complete taxon sampling and a 756‐gene matrix (Vargas *et al*., 2020). Neotropical *Costus* flowers exhibit wide variation in colors, color patterning and morphology, and were traditionally classified according to bee‐ and hummingbird‐pollination syndromes in early monographs (Maas, 1972, 1977). Many bee‐pollinated flowers show some red coloration (Kay & Schemske, 2003), and hummingbird‐pollinated *Costus* flowers tend to be shorter than bee‐pollinated *Costus* flowers (Yost & Kay, 2009), superficially contradicting some of the traits that are traditionally associated with pollination syndromes. Field pollination studies have shown that bee‐pollinated *Costus* are consistently specialized on orchid bees (Apidae: Euglossini) and hummingbird‐pollinated *Costus* are often specialized on hermit hummingbirds (Phaethornithinae: Trochilidae; Kay & Schemske, 2003). Orchid bees visit *Costus* for nectar and pollen, rather than the scents they collect from orchids and other sources, whereas hummingbirds visit *Costus* for nectar (Stiles, 1978b; Dressler, 1982; Kay & Schemske, 2003; Roubik & Hanson, 2004). Thus, *Costus* provides a unique and promising system in which to explore the evolution and convergence of hummingbird pollination.

We first review field pollinator observations to investigate how specialized Neotropical *Costus* are on bees vs hummingbirds. We then use a machine learning approach trained on floral traits of observed species to determine which morphological and color traits best predict pollination systems, and we use traits to predict pollination for all species. Next, we reconstruct ancestral states of pollination systems on a time‐calibrated *Costus* phylogeny to estimate how many pollination shifts have occurred and whether they are biased in direction. Across independent shifts to hummingbird pollination, we investigate whether evolution proceeds to convergent trait optima. We use our results to test common hypotheses for the directional bias from bee to hummingbird pollination, and we test the longstanding hypothesis that the evolution of hummingbird pollination is promoted in high‐elevation tropical environments using estimates of species ranges and climatic niches.

## Materials and Methods

### Study system

*Costus* L. is a pantropical genus of perennial monocot herbs with a species‐rich Neotropical clade nested within the relatively species‐poor African taxa (Maas‐van de Kamer *et al*., 2016; Vargas *et al*., 2020). The Neotropical spiral gingers comprise *c*. 59 named species plus several in various stages of taxonomic revision. They are found from sea level to cloud forests throughout tropical Central and South America. The phylogeny of Neotropical *Costus* was recently resolved with high support using a 756‐gene targeted sequencing approach (Vargas *et al*., 2020). In the present study, we use the fossil‐calibrated species tree from the concatenated dataset, in which monophyletic species are pruned to single representatives (fig. 4 in Vargas *et al*., 2020). The molecular clock dating estimates a crown age of 3 Ma (95% confidence interval 1.5–4.9 Ma) for the Neotropical radiation. Previous pollination studies in Neotropical *Costus* have shown that the melittophily and ornithophily pollination syndromes described by Maas (1972, 1977) reflect specialization on orchid bees and hummingbirds, respectively (Kay & Schemske, 2003). A preliminary ancestral state reconstruction indicated 11 transitions to hummingbird pollination (Vargas *et al*., 2020).

### Pollinator observations

We collated published and unpublished pollinator observation data (Table S1). For each species in the phylogenetic study, we recorded whether it had been observed, the total number of visits observed (if known), the proportion of observed visits that were by hummingbirds, the proportion of hummingbird visits that were by hermit hummingbirds, the proportion of visits matching the assigned pollination syndrome and the data source. Pollinator visits were defined as flower visits in which there was probable contact between the visitor and the anthers and stigma. Illegitimate visits in which nectar or pollen was removed without causing pollination were excluded. When a published source noted that a species was observed being pollinated by either hummingbirds or bees but did not present quantitative data, we recorded it as a single observation. We first investigated whether the pollination syndromes assigned in taxonomic treatments predict the most frequent pollinators. This type of analysis was previously done using observations of 11 species (Kay & Schemske, 2003), but here we included 28 *Costus* species. We also investigated whether the proportion of visits matching the most frequent pollinator type differed between bee‐ and hummingbird‐pollinated taxa using both ANOVA and phylogenetic ANOVA (*aov.phylo* in Geiger; Harmon *et al*., 2008).

### Floral traits

**Fig. 1:**
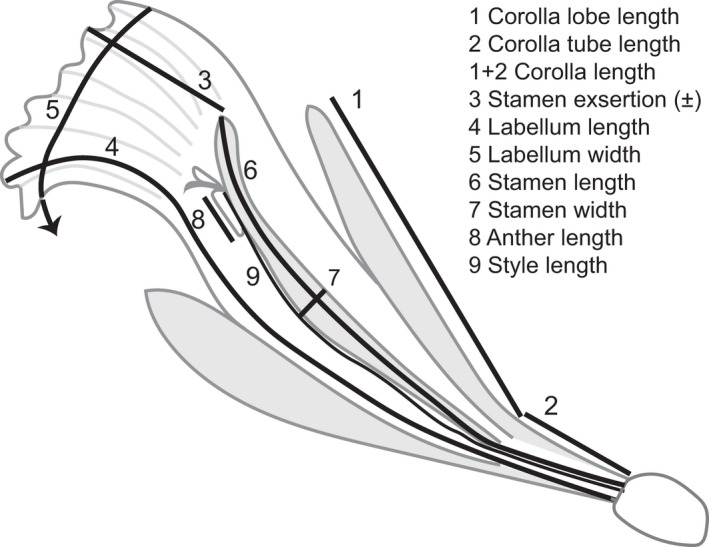
Diagram of a longitudinal cross‐section of a *Costus* flower showing morphological trait measurements. The flower is shown in gray and the trait measures are shown with black lines, except for the style, which is shown directly in black and was measured by laying it flat. Gray shading was added to offset flower parts for clarity. The arrow for the labellum width indicates that the entire labellum width could not be shown on a cross‐section. On an intact plant, the corolla tube and ovary are hidden by the subtending floral bract.

We gathered a dataset of morphological and color traits from live plants, taxonomic publications and well‐documented photographs (Fig. 1; Table S2). Live plants were measured in the UCSC glasshouses and field with digital calipers. The flower was first removed intact from the inflorescence. Corolla measures were taken from the dorsal petal, and the corolla was then removed to reveal the floral tube, which consists of a single fleshy stamen and a labellum. Stamen exsertion was measured as the distance the stamen tip protruded beyond the labellum and was negative when the stamen was inserted into the floral tube. Labellum and stamen size measurements were taken from dissected flowers in which the parts were laid flat. The gynoecium was removed from the rest of the flower and the style was laid flat and measured from the ovary to the base of the stigma.

We combined these with compatible measurements from monographs and protologues of *Costus* (Maas, 1972, 1977; Garcia‐Mendoza, 1991; Maas & Maas‐van de Kamer, 1997; Maas‐van de Kamer *et al*., 2016) for species sampled in Vargas *et al*. (2020). When taxonomic publications described a trait range for a species, we took the midpoint value.

To accommodate various data sources, we used categorical assignments of color based on human perception. Color descriptions were simplified into a series of categorical traits with a limited number of flower colors, including white, red, orange, yellow, green and purple. We scored the color of various flower parts, including the exposed portion of the floral bract, the corolla (dorsal petal), the primary labellum color (avoiding nectar guides), the main portion of the stamen and the stamen tip. We scored two binary characters for nectar guides – whether the labellum had red stripes, and whether the labellum had yellow stripes. Red stripes typically occur around the periphery of the labellum, whereas yellow stripes are typically restricted to the center of the labellum and align with the path bees follow when entering the flower.

Our study omitted certain traits that may be important for pollination but are not noted in taxonomic treatments based on herbarium specimens or are not observable from photos. For example, we did not include nectar reward, floral scent, quantitative color reflectance, or differences in the texture or stiffness of flowers, which all may be important in pollinator attraction and efficiency.

### Assembling the trait dataset

We assessed whether we could combine data from measurements of fresh flowers with measurements from the taxonomic literature by amassing data for 21 species from both sources. For continuous traits, we analyzed Pearson correlations and linear model slopes and intercepts between data sources. For all traits except stamen and labellum lengths, correlations were high (> 0.8). Stamen and labella lengths had been measured from the apex of the ovary on fresh material but from the apex of the fused corolla tube in the monographs. Therefore, we corrected the monograph values of stamen and labellum length by adding corolla tube length. In addition, stamen exertion was not reported in the taxonomic literature, so we calculated it as stamen length minus labellum length. For categorical color traits, there were no mismatches in the data between sources.

We combined data sources to arrive at species means for continuous traits and modes for categorical traits. In the glasshouse, when possible, we measured multiple flowers from a plant, multiple plants from a particular site and plants from multiple sites representing a particular species. We combined the data by first averaging across flowers per individual, then across individuals for a site and then across sites for a species. To combine data sources, we prioritized data from fresh material and high‐quality photographs and filled in missing data from the taxonomic literature. The combined dataset comprised 52 species, with 43 measured from fresh material and/or photographs and data for nine taken from the taxonomic literature.

### Estimating pollinators and importance of floral traits using machine learning

We used machine learning algorithms (Random Forest analysis, RF) to characterize and predict pollination systems following Dellinger *et al*. (2019, 2021). First, we trained and validated RF models on 28 *Costus* taxa with empirically documented pollinators. We calculated 1000 RFs, each consisting of 500 trees with four characters tested at each split. To quantify the robustness of the RF predictions, we calculated the percentage of instances where a taxon was correctly classified in the training models (*N* = 1000). The importance of each floral character (*N* = 17) for predicting documented primary pollinators was ranked by the mean decrease in Gini index over all 1000 RFs. This index is a measure of how important a floral character is for estimating pollinators across all the trees that make up an RF. Finally, we estimated pollinators for 21 taxa missing pollinator observations using the 1000 RFs above.

### Reconstructing ancestral states and biases in the direction of pollination shifts

Using all taxa with observed and inferred pollinators (*N* = 52), ancestral pollination states of pollinator were estimated using maximum likelihood under three separate models: equal rates, all rates different, and unidirectional rate from bee to bird with no reversals. Models were fit with the *fitDiscrete* function in Geiger (Harmon *et al*., 2008). Model selection was then used to determine the optimal model using the *aic.w* function in Phytools (Revell, 2012). Using the optimal model, ancestral states were then estimated using 1000 iterations of Bayesian stochastic character mapping using the *make.simmap* function in the Phytools package. Finally, to determine if our results were driven by inferred pollination (which differed somewhat from syndromes assigned in taxonomic treatments), this analysis was repeated using syndromes assigned in taxonomic treatments, and only those tip taxa with pollinator observations (*N* = 28).

Directional or irreversible character state changes can be difficult to assess, especially if root character states are misassigned or speciation or extinction rates are state‐dependent (Goldberg & Igić, 2008). We are confident in assigning the root of the Neotropical radiation as bee‐pollinated because African *Costus* relatives predominantly have bee‐pollination syndromes and there are no hummingbirds in Africa. Although one African *Costus* species is reported as having a passerine pollination syndrome, phylogenetic evidence supports it as being independently evolved (Maas‐van de Kamer *et al*., 2016). In addition, we performed a Binary State Speciation and Extinction (BISSE) analysis using the Diversitree package to determine whether there were significant pollination‐dependent differences in diversification or transition rates (Maddison *et al*., 2007; FitzJohn, 2012). We ran the Markov chain Monte Carlo (MCMC) chain for 10 000 steps and, because our tree is small for a BISSE analysis, generated priors using the *make.prior.exponential* function as recommended in Diversitree.

### Examining convergence in pollination

Because many of the 17 floral traits were highly correlated, we first reduced the dimensions of our trait dataset. We estimated multivariate phenotypes using factor analysis of mixed data in the R package factominer (Lê *et al*., 2008). To run the factor analysis, we first imputed 34 missing data points across 10 continuous traits using the regularized algorithm and five components in the missMDA package (Josse & Husson, 2016). No data were missing for the seven categorical traits. Imputed data composed 3.8% of our dataset. We then performed factor analysis on the complete dataset of 17 floral traits for 52 taxa using the FAMD function and visualized the output with the factoextra package (Kassambara & Mundt, 2020). We output coordinates in the first 10 dimensions for each taxon for use in further analyses.

We visualized the evolution of floral traits by projecting the phylogeny on species' values for dimensions 1 and 2 using Phytools (Revell, 2012). We then investigated whether floral dimensions 1–10 were predicted by pollination state using both ANOVA and phylogenetic ANOVA (aov.phylo; Harmon *et al*., 2008). Given the significant relationship between dimension 1 and pollination, we assessed patterns of convergent evolution of dimension 1 under an Ornstein–Uhlenbeck process using the l1ou R package (Khabbazian *et al*., 2016; for a similar approach, see Smith & Kriebel, 2018). We note that l1ou does not require an a priori designation of where regime shifts (i.e. shifts in adaptive optima) occurred and instead estimates both the phylogenetic placement and magnitude of shift (function estimate_shift_configuration), and whether shifts are towards one or multiple regimes (function estimate_convergent_regimes). Convergence is inferred as independent shifts to the same regime.

### Assessing whether pollination system varies by elevation and climate

Bees are thought to be less efficient pollinators at high elevations due to cool wet conditions whereas hummingbirds are efficient across all elevations (Cruden, 1972; Armbruster & McCormick, 1990; Dellinger *et al*., 2021). Therefore, we hypothesized that bee‐pollinated taxa should occupy lower elevations that are warmer and drier on average, and have lower variance in these attributes, than hummingbird‐pollinated taxa. To estimate the median elevation of each taxon, we used a set of previously published cleaned occurrence records (Vargas *et al*., 2020) and extracted elevation associated with each unique occurrence based on latitude and longitude (R package elevatr function ‘get_elev_raster’; Hollister *et al*., 2020). This resulted in 3772 unique occurrences for 47 taxa (average 80 per taxon, range 4–500). Climate niche estimates were used directly from Vargas *et al*. (2020), where principal component analysis summarized four climate variables for all unique occurrences: mean annual temperature, mean annual precipitation, temperature seasonality and precipitation seasonality. Climate PC1 primarily captured variation in precipitation and the seasonality of temperature and precipitation, whereas PC2 primarily captured variation in temperature. The niche position of each species was then estimated as the mean of PC1 and PC2. To test whether median elevation, climate PC1 and climate PC2 were predicted by pollination, we used phylogenetic ANOVA in three separate models (*aov.phylo*; Harmon *et al*., 2008). To test whether the variance in median elevation, climate PC1 and climate PC2 differed by pollination, we used three separate Levene's tests (leveneTest function, car package in R; Fox & Weisberg, 2019). Ideally, we would account for shared ancestry in the Levene's test, but this has yet to be implemented in R to our knowledge.

## Results

### Pollination syndromes predict primary pollinators

**Fig. 2:**
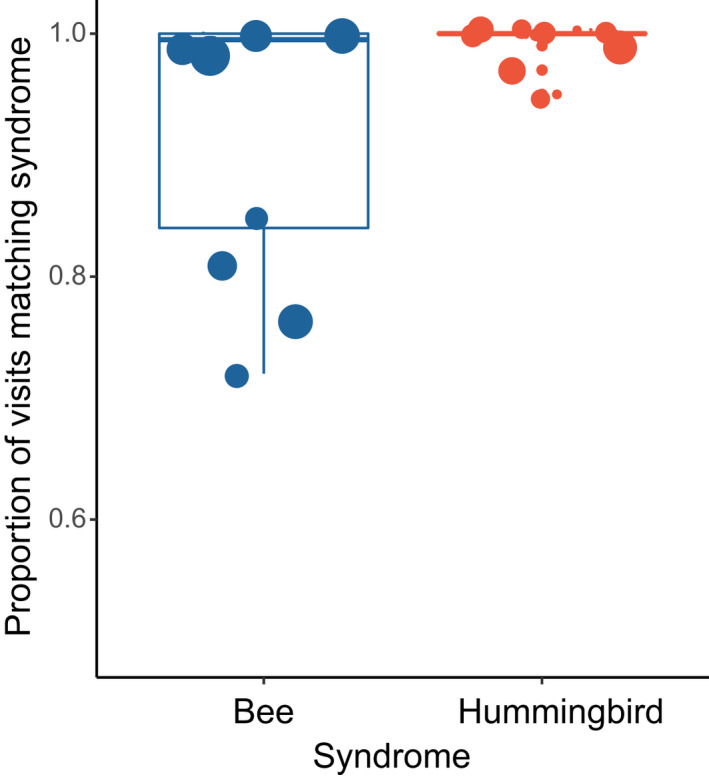
The proportion of pollinator visits matching the described pollination syndrome in Neotropical *Costus* is slightly lower on average for bee‐pollinated species compared to hummingbird‐pollinated species, although in all cases over 70% of visits matched the syndrome prediction. Points are colored by syndrome and proportional in size to the natural log (+1) of the number of observations. Boxplots show the median, the first and third quartiles, and the most extreme values no further than 1.5× the interquartile range from the box.

Across 28 species with documented pollinator visits, pollination syndrome assignment in the taxonomic literature always predicted most pollinator visits (mean 97% of visits, range 72–100%). Bee‐pollinated species were primarily visited by orchid bees, although some bumblebee visits were also reported for one species (*Costus arabicus*; Bergamo *et al*., 2016), and hummingbird‐pollinated species were primarily visited by hermits (mean of 84% of hummingbird visits by hermit hummingbirds). For bee syndrome species, all the unpredicted pollinator visits were by hummingbirds, and for hummingbird syndrome species, all of the unpredicted pollinator visits were by bees. The proportion of visits matching the syndrome was higher for hummingbird syndrome species (*F*_1,26_ = 6.34, *P* = 0.018; Fig. 2), although this result was not significant when incorporating phylogeny (phylogenetic ANOVA, *P* = 0.14).

### A limited number of floral traits predict bee vs hummingbird pollination

**Fig. 3:**
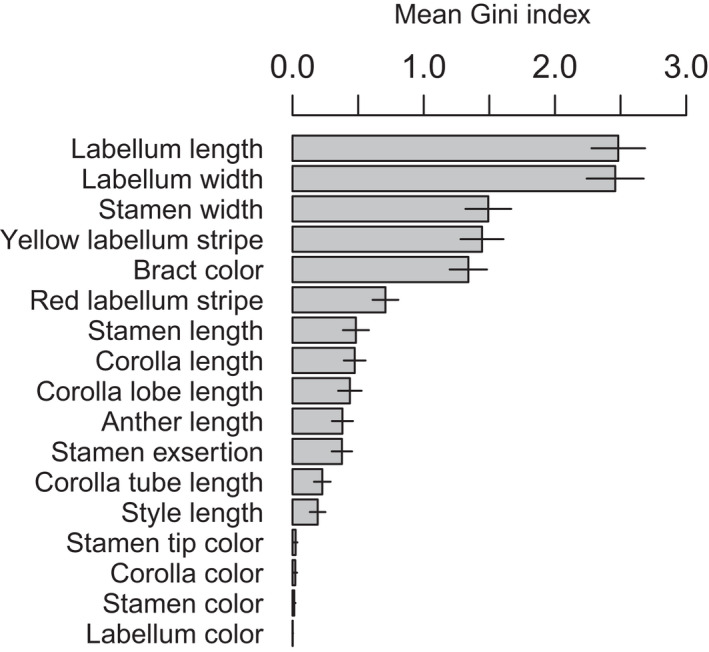
Ranked importance of measured floral traits at predicting bee vs hummingbird pollinator identity for the 28 Neotropical *Costus* with observed pollinators from the Random Forest (RF) analysis. Floral traits were then used to predict pollinators for unobserved *Costus* species. Error bars represent 1 standard deviation in Gini index values from 1000 RFs.

Overall, machine learning algorithms had high predictive accuracy across taxa with pollinator observations (*N* = 28): accuracy was 100% across 27 of 28 taxa, and one taxon, *C. erythrocoryne*, was accurately predicted in 80% of models. The top two most important floral traits for predicting pollinator identity were labellum length and width (Fig. 3), with bee‐pollinated taxa having labella that were 2.0 and 3.3 times the length and width of hummingbird‐pollinated taxa. Stamen width, which together with the labellum forms the floral tube, was the third ranked trait (on average 1.6 times greater for bee‐ relative to hummingbird‐pollinated taxa), followed by presence/absence of yellow labellum stripes, bract color and presence/absence of red labellum stripes. All bee‐pollinated taxa had yellow labellum stripes, and all but one had green bracts. By contrast, only one of 16 hummingbird‐pollinated taxa had yellow labellum stripes and none had green bracts (instead bracts were either red, orange or yellow). All except one bee‐pollinated taxon (*C. villosissimus*) had red labellum stripes, whereas a single hummingbird‐pollinated taxon was polymorphic for red labellum stripes (*C. wilsonii*). The main colors of the stamen, corolla and labellum were the least predictive traits.

For 21 taxa lacking pollinator observations, models predicted 10 to be bee‐ and 11 hummingbird‐pollinated. For 18 of these taxa, model predictions matched expert opinion (Maas, 1972, 1977; Vargas *et al*., 2020). For the three remaining taxa (*C. dirzoi*, *C. varzearum* and *C*. sp_nov19168), the model predicted hummingbird pollination whereas expert opinion was bee pollination.

### Transitions to hummingbird pollination may be irreversible

**Fig. 4:**
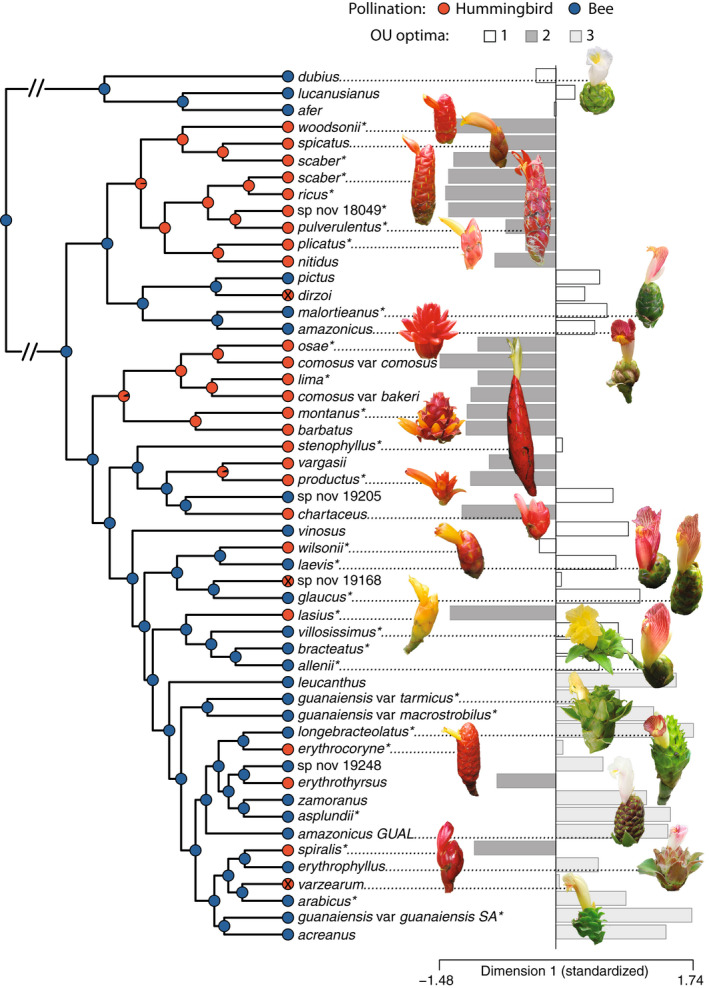
Time‐calibrated *Costus* tree with ancestral state reconstruction of pollination system, inflorescence photos for selected species and character states for floral trait dimension 1 Orenstein–Uhlenbeck (OU) optima. Species predicted to be hummingbird‐pollinated by Random Forest analysis but not in *Costus* taxonomic treatments are indicated with an ‘x’ in their tip state symbol. Asterisks indicate species for which pollinators have been documented in the wild (Table S1). Branch lengths are condensed between the African outgroups and the Neotropical radiation.

Model comparison determined an irreversible model of pollination system evolution to be optimal, in which Neotropical *Costus* species transition from bee to hummingbird pollination with no reversals (Fig. 4). Using the irreversible model, Bayesian stochastic character mapping then inferred a mean of 13.11 total transitions from bee to bird (range 13–15). These results regarding the irreversibility of shifts were not sensitive to the inferred pollination syndromes of tip taxa. Using syndromes assigned by taxonomic treatments and expert opinion, model comparison favored the irreversible model with 10.16 total transitions from bee to hummingbird inferred (range 10–12). The irreversible model was also favored using a reduced taxonomic dataset of only those taxa with pollinator observations (*N* = 28) with 7.45 total bee to hummingbird transitions inferred (range 7–9). Although BISSE analysis produced higher maximum‐likelihood estimates of speciation rates for bee‐ compared to hummingbird‐pollinated taxa (λ of 73.95 vs 46.78) and higher rates for bee‐to‐hummingbird transitions compared to hummingbird‐to‐bee transitions (*q* of 28.20 vs 0), the maximum‐likelihood model did not fit the data significantly better than constrained models with equal rates (likelihood‐ratio tests; speciation rates: *P* = 0.16; transition rates: *P* = 0.15; Fig. S1). Extinction rates were estimated as zero for both character states.

### Pollination systems show convergent phenotypic optima

**Fig. 5:**
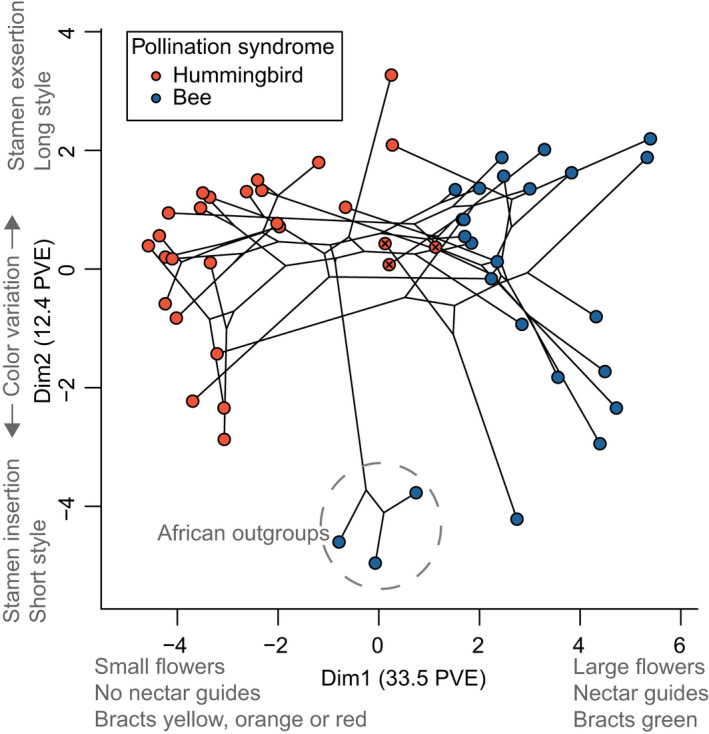
*Costus* phylogeny projected onto the first two dimensions of floral trait space, which explain 33.5% and 12.4% of the variation (PVE) in floral traits, respectively. Species predicted to be hummingbird‐pollinated by Random Forest analysis but not in *Costus* taxonomic treatments are indicated with an ‘x’ in their tip state symbol.

Using factor analysis of mixed data to reduce the dimensionality of the 17 floral traits resulted in two primary dimensions explaining 33.5% and 12.4% of the variation, respectively, across 52 species (Figs 5, S2). Dimension 1 was strongly correlated with pollination (*F*_1,50_ = 126.11, *P* < 0.001; phylogenetic ANOVA, *P* < 0.001), with large values representing species with large flowers, nectar guides and green bracts (i.e. bee pollination) and small values representing species with small flowers, no nectar guides, and bracts that were either yellow, orange or red (i.e. hummingbird pollination). Bee‐ and hummingbird‐pollinated taxa in the Americas show little overlap in dimension 1 (Fig. 5). By contrast, dimension 2 was not predicted by pollination (*F*_1,50_ = 2.30, *P* = 0.136; phylogenetic ANOVA, *P* = 0.368) and represents variation in primary flower colors, stamen exsertion and style length, all of which varied widely within both pollination systems. Like dimension 2, none of the other eight floral dimensions were predicted by pollination system (phylogenetic ANOVAs, *P* > 0.35).

Given the strong association between floral dimension 1 and pollination, we tested for significant optima shifts in dimension 1 across the phylogeny and for evidence of convergent evolution. We detected eight optima shifts total under an Orenstein–Uhlenbeck (OU) model (Figs 4, S3): six shifts were identified as convergent on small values of dimension 1 and were aligned with shifts towards hummingbird pollination, one shift was towards larger values of dimension 1 and was associated with a clade of large‐flowered South American bee‐pollinated taxa, and one shift was a reversal from small values (associated with hummingbird pollination) towards larger values (associated with the ancestral regime of bee pollination). We note that this final shift conflicts with the irreversible model of pollination system detected above. Overall, we identified three optima, two associated with bee pollination and one associated with hummingbird pollination, although the associations were imperfect (see the Discussion section).

### Hummingbird pollination is not associated with higher elevation or cooler, wetter climates than bee pollination

**Fig. 6:**
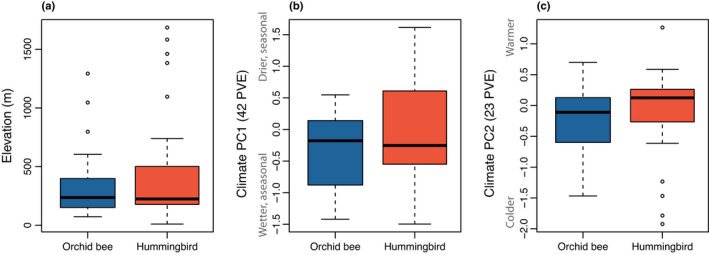
Neotropical *Costus* species with orchid bee vs hummingbird pollination systems do not differ by median elevation (a), climate niche PC1 (b) or climate niche PC2 (c). Climate niches are circumscribed in Vargas *et al*. (2020). Boxplots show the median, the first and third quartiles, and the most extreme values no further than 1.5× the interquartile range from the box.

The median elevation for 22 bee‐ and 25 hummingbird‐pollinated taxa ranged from 72 to 1293 m and from 10 to 1686 m respectively. There was no evidence of correlated evolution of pollination and elevation (phyANOVA *P* = 0.5654), and no evidence that the variance in median elevation differed by pollination (Levene's test, *F*_1,45_ = 1.40, *P* = 0.242; Fig. 6). Similarly, we found no evidence of correlated evolution of pollination and climate niche (phyANOVA: climate PC1, *P* = 0.445; climate PC2, *P* = 0.787), and no evidence that the variance in climate niche differed by pollination (Levene's test: climate PC1, *F*_1,45_ = 0.608, *P* = 0.440; climate PC1, *F*_1,45_ = 0.27, *P* = 0.606). Despite multiple transitions towards hummingbird pollination in *Costus*, there is no macroevolutionary evidence that shifts toward hummingbird pollination consistently occur at higher elevations or in cooler, wetter climates. Instead, hummingbird pollination occurs across the entire elevational range occupied by *Costus*.

## Discussion

Evolutionary transitions to hummingbird pollination are pervasive across disparate plant lineages throughout the Americas, and shed light on convergence, constraint and directionality. The well‐resolved Neotropical *Costus* phylogeny, extensive field pollination data and availability of flowering specimens allowed us to explore the evolution of hummingbird pollination and compare *Costus* to other study systems. *Costus* shows remarkable convergence across multiple independent transitions, and our data refute several common explanations for the prevalence of transitions from bee to bird pollination.

We first established the functional convergence of pollination syndromes by showing that taxonomically described syndromes accurately predict the most frequent pollinators in all 28 observed *Costus* species. Compared to bee flowers, hummingbird flowers have smaller floral tubes formed by short narrow labella and narrow stamen, the labella lack an expanded landing surface and nectar guides, and the flowers are subtended by brightly colored bracts. The consistency of these traits enabled confident predictions of pollinators for unobserved species, which in turn facilitated ancestral state reconstruction and explicit phylogenetic analysis of convergence.

Remarkably, we found a single phenotypic optimum shared across most independent transitions to hummingbird pollination, despite transitions occurring across a broad temporal and geographic range (Vargas *et al*., 2020). *Costus* are substantially specialized on orchid bees and hermit hummingbirds, which are both known for traplining foraging behavior among widely dispersed nectar‐rich flowers (Janzen, 1971; Stiles, 1978a; Stiles & Wolf, 1979). Hermits and orchid bees also tend to have long bills and proboscides relative to other hummingbirds and bees, respectively. The similarity in nectar reward, flower sizes and foraging patterns between these specific groups of bee and hummingbird pollinators may facilitate transitions, and the specialization on hermits may contribute to the striking levels of convergence seen in hummingbird flowers.

Interestingly, we found less convergence among bee flowers, despite bee pollination being the shared ancestral condition. There were two ‘bee’ optima, with smaller bee flowers primarily found in Central America and larger bee flowers occurring in a clade of South American species distributed in the Amazon, the lower eastern Andes and the Chocó ecoregion. The latitudinal gradient in orchid bee diversity and species composition may explain these optima. Amazonian and Andean communities tend to comprise larger‐bodied representatives of *Euglossa* plus many species in the larger‐bodied genera *Eulaema*, *Exaerete* and *Eufriesea*, whereas northern portions of the orchid bee range are dominated by smaller‐bodied *Euglossa* (Dressler, 1982; Cameron, 2004; Roubik & Hanson, 2004). Field studies associating *Costus* flowers with particular bee morphologies are underway to test the functional significance of flower size variation.

*Costus* floral traits are not perfectly bimodal between bee and hummingbird pollination, and intermediates may shed light on how evolutionary transitions proceed. For example, three taxa predicted to be bee‐pollinated in monographs but predicted to be hummingbird‐pollinated here are all small‐flowered but with typical bee coloring. This discrepancy could indicate generalized pollination or transitional phenotypes between pollination systems. Flower size may decrease as an early step towards hummingbird pollination. Alternatively, they may live in geographic regions or habitats with atypical pollinators. Two occur near the northern range limit of *Costus* where only small‐bodied orchid bees occur (Burquez, 1997; Roubik & Hanson, 2004). These three species are high priorities for field pollinator observations.

A second source of overlap between bee and hummingbird flowers comes from analyses of OU optima. Specifically, the single hummingbird optimum does not include some species known to be hummingbird‐pollinated. Those species share the optimum of the most closely related bee‐pollinated species and are singletons (i.e. a single member of a clade), indicating a pollination shift at a tip in the phylogeny. Two of these species have exceptionally long flowers, and one has short flowers polymorphic for nectar guides on its relatively wide labellum. These species are potentially fruitful systems for studying the early stages of hummingbird adaptation. However, other hummingbird‐pollinated singletons share the ‘hummingbird’ optimum (*C. lasius*, *C. erythrothyrsus*, and *C. spiralis*), so singletons do not consistently exhibit intermediate floral traits. In addition, the three species predicted to be hummingbird‐pollinated by our models but bee‐pollinated in the taxonomic literature do not share the ‘hummingbird’ optimum. As discussed above, they are all small and narrow relative to other bee flowers, although they exhibit typical bee colors. Thus, although there may be intermediate forms in *Costus*, they show a variety of phenotypes and some may be explained by other factors such as local pollinator assemblages.

In comparison with other clades with analogous bee to hummingbird transitions, *Costus* shows some surprising floral traits. Hummingbird flowers are consistently smaller compared to bee‐pollinated relatives. This contrasts with several temperate (*Penstemon*, *Aquilegia*, *Mimulus*: Wilson *et al*., 2004; Whittall & Hodges, 2007; Grossenbacher & Whittall, 2011) and tropical or subtropical (*Ruellia*, *Iochroma*, *Salvia*: Tripp & Manos, 2008; Smith & Kriebel, 2018; Benitez‐Vieyra *et al*., 2019) plant clades, but aligns with syndromes in Gesneriaceae, in which bee flowers are often visited by orchid bees (Dressler, 1982; Serrano‐Serrano *et al*., 2017, and references therein). The difference in the direction of size evolution may be unique to orchid bee pollination. In *Costus* and other orchid bee‐pollinated plants, the relatively large‐bodied bees crawl inside a large distal floral chamber, or ‘gullet’, and use their long proboscides to access a narrow, nectar‐filled base (Dressler, 1982). Another contrast with other plant clades is the lack of importance of primary flower colors of the petals or labellum. Primary flower colors vary widely within both pollination systems and loaded highly on floral trait dimension 2, which was not correlated with pollination system. The quintessential red coloration of hummingbird flowers may be more common in temperate hummingbird‐pollinated plants. Nevertheless, *Costus* show a typical narrowing of the flower to produce a tube capable of efficiently transferring pollen to a hummingbird bill.

We found no support for the longstanding hypothesis that cool, wet, tropical montane conditions drive adaptation to bird pollination (Cruden, 1972). Our results differ from those for Melastomataceae, in which vertebrate‐syndrome taxa tend to occur at higher elevations relative to bee‐syndrome taxa (Dellinger *et al*., 2021). The difference between clades may be explained by the specialization of *Costus* on orchid bees, which reach their highest diversity (although not necessarily abundance) in cloud forests (Cameron, 2004; Roubik & Hanson, 2004), although the cool wet conditions do restrict diel foraging activity (Armbruster & McCormick, 1990; Armbruster & Berg, 1994). Moreover, *Costus* may simply not reach high enough elevations for this process to play out, rarely occurring above 2000 m. Nevertheless, hummingbird pollination regularly evolves in lowland *Costus*, suggesting advantages to hummingbird pollination that do not require invoking high elevation and a concomitant paucity of bees. Our results contradict the ‘tropical flip’ pattern in which vertebrate pollination dominates higher elevations in the tropics but not in temperate regions (Dellinger *et al*., 2022a).

We found evidence in *Costus* for a directional bias in transitions to hummingbird pollination. Similar directional biases have been documented in multiple plant clades, including *Penstemon*, *Aquilegia*, Antirrhineae and *Mimulus* (Whittall & Hodges, 2007; reviewed in Rosas‐Guerrero *et al*., 2014; Wessinger *et al*., 2016; Ogutcen *et al*., 2017; Nelson *et al*., 2021). The few clades showing reversals include *Ruellia* (Acanthaceae; Tripp & Manos, 2008), Gesneriaceae (Serrano‐Serrano *et al*., 2017), Iochrominae (Smith & Kriebel, 2018), Loasaceae subfam. Loasoideae (Ackermann & Weigend, 2006) and *Salvia* (Kriebel *et al*., 2020), mostly Neotropical clades with highly labile and diverse pollination systems. Nevertheless, all of these clades except Iochrominae show a strong bias in the direction of pollinator shifts from insect to bird. Thus, biases in direction are pervasive across disparate plant clades and beg a common explanation.

Our results can address some of the many hypotheses put forth to explain the prevalence of insect‐to‐bird shifts. First, the biased drive to hummingbird pollination is proposed to occur because of a bias towards the evolution of increasingly long flowers, either through Darwin's coevolutionary race (Darwin, 1862) or by pollinator shifts to increasingly long‐tongued pollinators, as proposed for *Aquilegia* (Whittall & Hodges, 2007). Our results directly contradict this hypothesis as derived hummingbird flowers are consistently shorter than bee flowers.

Second, hummingbird transitions are common in *Costus* even though they do not show the same violet to red floral pigment transitions seen in *Penstemon*, *Ipomoea* and *Aquilegia*, directly contradicting the idea of constraints imposed by anthocyanin evolution (Rausher, 2008). In *Costus*, red pigments are pervasive, and it is the location and/or timing of color expression that differs. For example, most bee flowers have elaborately striped red nectar guides around the labellum, whereas red tends to be evenly distributed throughout hummingbird flowers. Moreover, all *Costus* species display red on the interior of the bracts upon fruit ripening, whereas hummingbird‐pollinated species often display red on the exterior of the bracts during flowering. Also, many hummingbird‐pollinated *Costus* flowers are not red and instead display a range of colors, including white, yellow and orange. This color diversity may be a feature of tropical hummingbird flowers, since temperate flowers are thought to be under strong convergent selection for red to attract limited species of migratory hummingbirds (Grant & Grant, 1968), whereas tropical flowers may compete for many resident hummingbird species. For example, Muchhala *et al*. (2014) found flower color to be overdispersed among sympatric hummingbird‐pollinated Andean Iochrominae (Solanaceae), suggesting interspecific competition for pollinators.

Third, hummingbird pollination might be expected to be the derived condition because hummingbirds diversified more recently than bees, with a crown‐group age of 22 Ma (McGuire *et al*., 2014) vs 123 Ma for bees (Cardinal & Danforth, 2013) or 34–38 Ma for orchid bees (Ramírez *et al*., 2011). However, the entire Neotropical *Costus* radiation has taken place in the last *c*. 3 million years, well after hummingbirds underwent their major radiation (Abrahamczyk & Renner, 2015). Moreover, hummingbird pollination evolved in *Costus* shortly after the *Costus* crown radiation in the Americas, with two shifts estimated between 2 and 2.5 Ma (Vargas *et al*., 2020). As many transitions have occurred since that time, it is difficult to invoke time as limiting reversals to bee pollination.

Other ideas regarding the prevalence of transitions to hummingbird pollination are not contradicted by our study. For example, hummingbirds may generally be difficult to exclude from flowers with nectar, leading them to be common visitors to a wide variety of flowers and providing an entry point for adaptation to hummingbird pollination. In bee‐pollinated *Costus*, hummingbirds are fairly common secondary pollinators (Fig. 1). Hummingbirds seem to lack innate color preferences and visit any flower with an accessible nectar reward (Stiles, 1976). The nectar reward in bee‐pollinated *Costus* is similar in volume and concentration to that in hummingbird‐pollinated *Costus*, so opportunistic visits are expected, especially when flower resources are limited (Stiles, 1978b; Kay & Schemske, 2003). By contrast, features of hummingbird flowers, such as red coloration and a lack of nectar guides or landing platform, are known to effectively exclude bees (Schemske & Bradshaw, 1999; Castellanos *et al*., 2004; Thomson & Wilson, 2008; Bergamo *et al*., 2016), and may therefore limit reversals to bee pollination.

Finally, hummingbird pollination may represent a generally more effective pollination system. Birds can travel long distances between plants and, unlike bees, do not groom or consume pollen (Castellanos *et al*., 2003; Thomson & Wilson, 2008; Krauss *et al*., 2017). Previous studies of *Costus* pollination found higher visitation rates by bees but did not examine pollen transfer efficiency or pollen quality (Kay & Schemske, 2003). Other studies have found more extensive gene flow in vertebrate‐pollinated compared to bee‐pollinated plants (Krauss *et al*., 2017; Dellinger *et al*., 2022b). Outcrossing via hummingbirds could benefit *Costus* species, which, although self‐compatible, have substantial heterozygosity and inbreeding depression (Schemske, 1983; Surget‐Groba & Kay, 2013). On the other hand, orchid bees are known to forage over long distances and may not have similar effects on breeding patterns as most bees (Janzen, 1971; Janzen, 1981; Ackerman *et al*., 1982; but see Opedal *et al*., 2017). This hypothesis remains to be explored in Neotropical *Costus*.

## Author contributions

KMK and DLG designed the research, performed the research, collected, analyzed and interpreted the data, and wrote the manuscript.

## Supporting information


**Fig. S1** Binary State Speciation and Extinction model posterior probability distributions for speciation and extinction rates.
**Fig. S2** Factor analysis biplots of 17 floral traits for 52 *Costus* taxa.
**Fig. S3** Visualization of floral optima shifts under an Orenstein–Uhlenbeck model of trait evolution in neotropical *Costus*.

**Table S1** Pollinator observations and syndrome classifications of 52 *Costus* taxa.
**Table S2** *Costus* floral trait data sources.
Please note: Wiley Blackwell are not responsible for the content or functionality of any Supporting Information supplied by the authors. Any queries (other than missing material) should be directed to the *New Phytologist* Central Office.

## Data Availability

Data and custom scripts can be accessed in Dryad at doi: 10.7291/D1C39G.
