# Supplementary material for: Evolutionary convergence on hummingbird pollination in Neotropical Costus provides insight into the causes of pollinator shifts
Source: New Phytol. 2022 Sep 24;236(4):1572–83. doi: 10.1111/nph.18464 (PMC9826479; doi:10.1111/nph.18464)
Supplement: Supplementary file 1 — Fig. S1 Binary State Speciation and Extinction model posterior probability distributions for speciation and extinction rates. Fig. S2 Factor analysis biplots of 17 floral traits for 52 Costus taxa. Fig. S3 Visualization of floral optima shifts under an Orenstein–Uhlenbeck model of trait evolution in neotropical Costus. [file NPH-236-1572-s001.pdf]

***New Phytologist* Supporting Information**

Article title: Evolutionary convergence on hummingbird pollination in Neotropical *Costus* provides insight into the causes of pollinator shifts

Authors: Kathleen M. Kay and Dena L. Grossenbacher

Article acceptance date: 10 August 2022

The following Supporting Information is available for this article:

Fig. S1 BISSE model posterior probability distributions for speciation and extinction rates

Fig. S2 Factor analysis biplots of 17 floral traits for 52 *Costus* taxa

Fig. S3 Visualization of floral optima shifts under an Orenstein-Uhlenbeck model of trait evolution

Table S1 Pollinator observations and syndrome classifications of 52 *Costus* taxa

Table S2 Floral trait data sources

Fig. S1 BISSE model posterior probability distributions for speciation and extinction rates of bee- and hummingbird-pollinated Neotropical *Costus*. Likelihood-ratio tests (LRTs) of model comparisons were used to test hypotheses that rates differ for pollination states. For each type of rate, the maximum likelihood model did not fit the data significantly better than a constrained model with equal rates between pollination states.

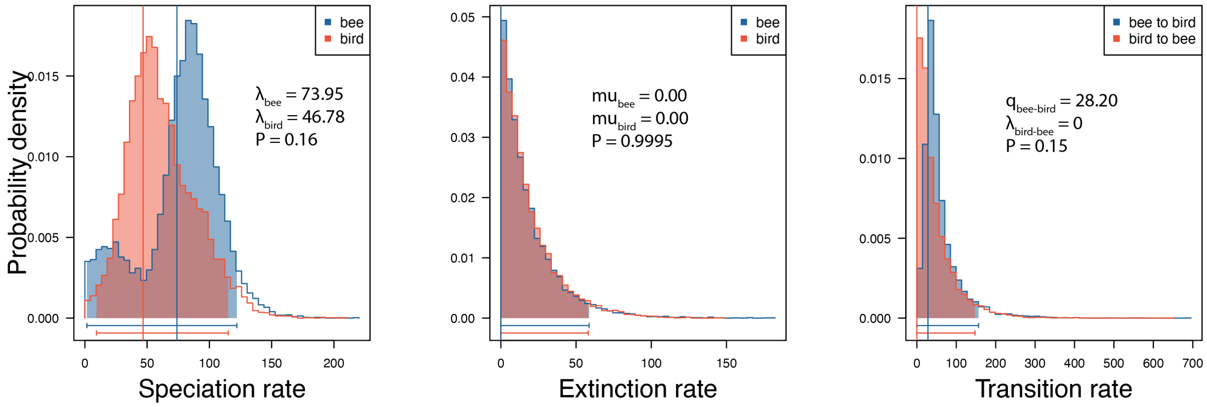

Fig. S2 Factor analysis loadings of 17 traits on the first two dimensions, explaining 33.5 and 12.5% respectively of variation in floral traits among 52 *Costus* taxa. (**A**) Loading scores of 10 continuous traits (**B**) Loading scores of 10 continuous and 7 categorical traits, with categorical traits in red text (**C**) Biplot with representations of the categories for 7 categorical traits and scores for all 52 taxa (labelled 1-52).

A. Quantitative trait loadings

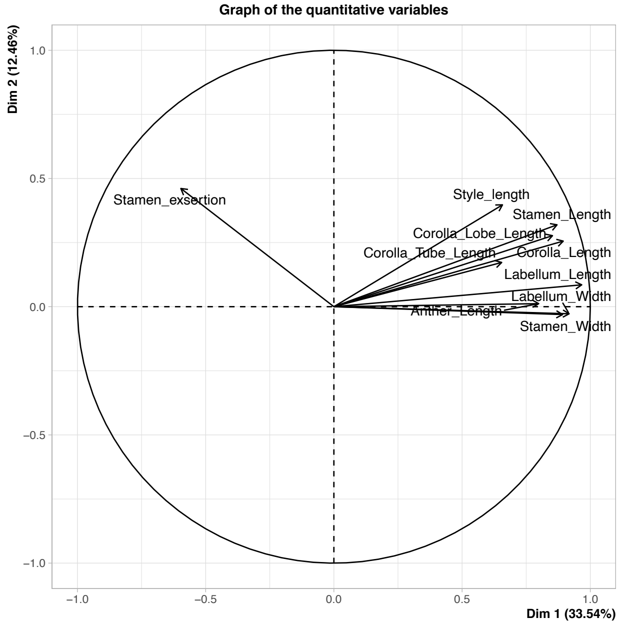

B. Quantitative and discrete trait loadings

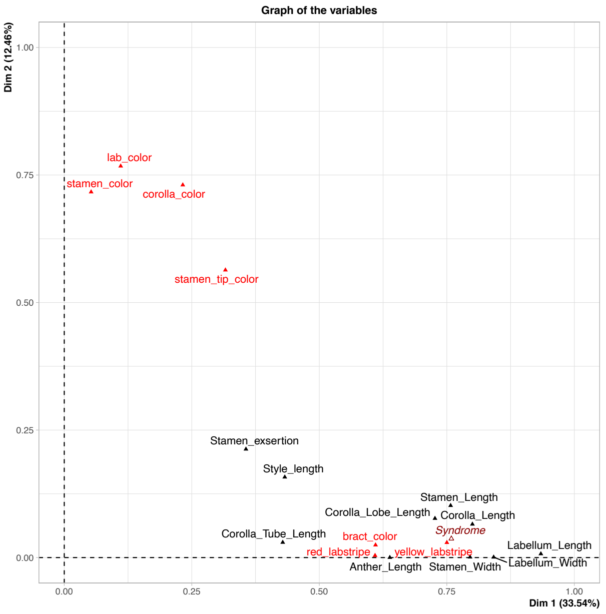

C. Biplot of scores for all 52 species and discrete trait loadings

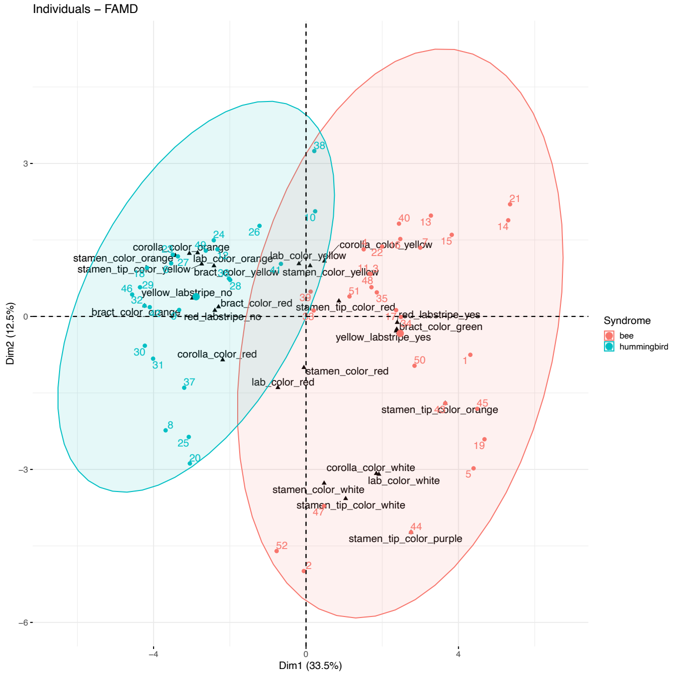

Fig. S3 Estimated shifts in phenotypic optima of floral dimension 1 for Neotropical *Costus*. Locations of shifts in adaptive optima are indicated with asterisks with adjacent values representing the direction and magnitude of the shift. Branches with equivalent optima have the same branch color: grey represents the likely ancestral optima associated with bee pollination syndrome; blue represents a novel optima shift towards smaller dimension 1 values associated with hummingbird pollination syndrome; green represents a single novel optima shift towards larger bee pollinated syndrome flowers. The histogram on the right indicates standardized floral dimension 1 values ((trait – mean)/standard deviation) for each species, colored according to the three OU optima.

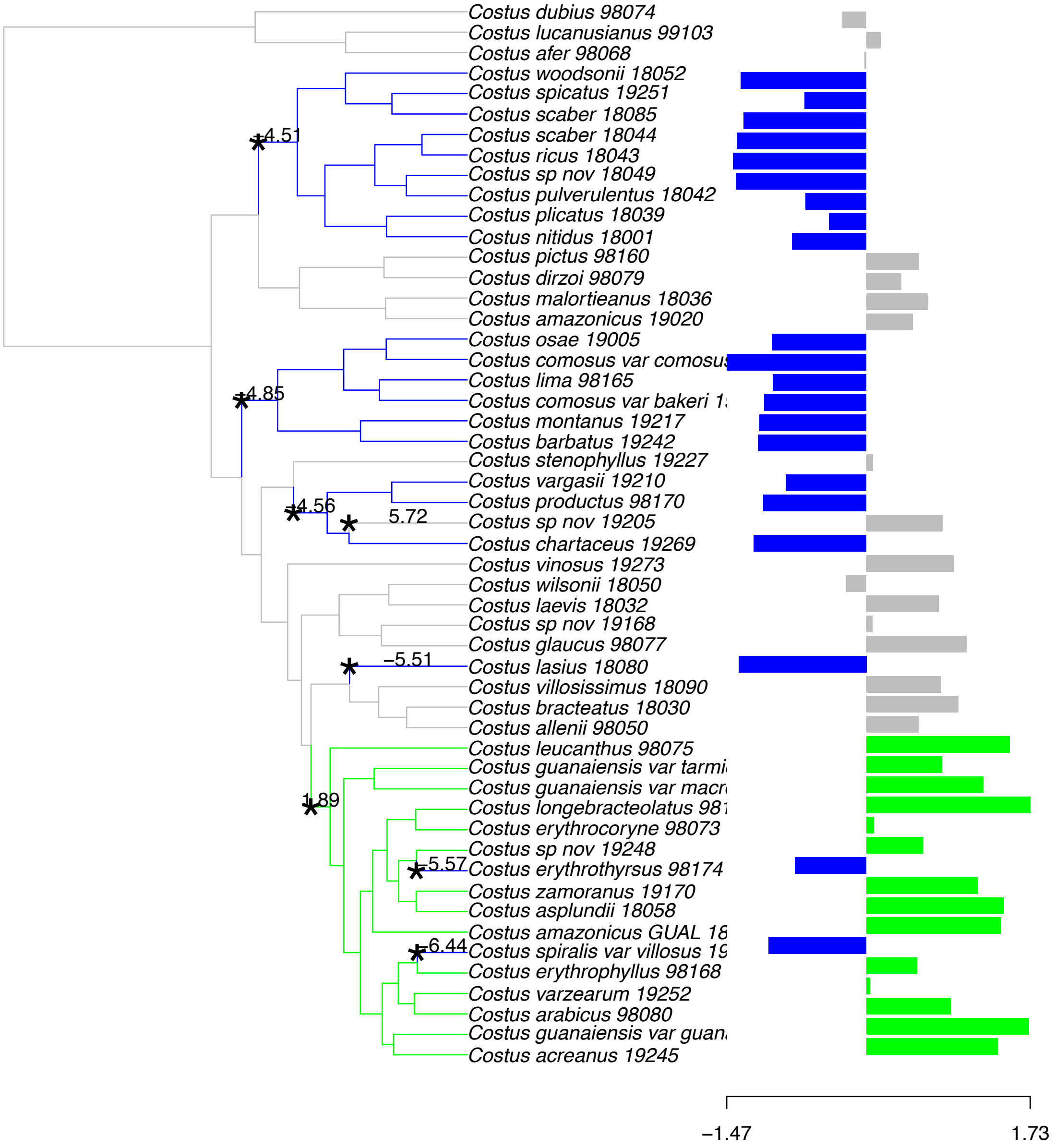

Table S1 Data file of pollinator observations and syndrome classifications of 52 *Costus* taxa. The following columns are included: taxon identity (tip_label), pollination syndrome as determined by taxonomic treatments and our quantitative analyses (syndrome), indication of whether pollinator observations have been made for a given taxon (observed), proportion of floral visitors that were hummingbirds (prop_hummingbird), proportion of floral visitors that were hermit hummingbirds (prop_hermit), total number of floral visitors observed (Total_visits_observed), proportion of floral visitors that match the pollination syndrome (prop_matching), source of pollinator observation data (source), additional notes regarding pollinator observation data (notes). A .csv version of this table is available at https://doi.org/10.7291/D1C39G

Table S2 Data file of sources used for gathering continuous and discrete floral trait data for 52 *Costus* species. The following columns are included: taxon identity (tip_label) and sources (data_sources). A .csv version of this table is available at https://doi.org/10.7291/D1C39G
